# Does style investing uniformly affect correlations in small and large markets?

**DOI:** 10.1016/j.heliyon.2020.e04881

**Published:** 2020-09-22

**Authors:** Valentina Galvani

**Affiliations:** Economics Department, University of Alberta, Canada

**Keywords:** Style investing, Risk aversion, Correlations, Risk diversification, Country exchange traded funds, Finance, Financial crisis, Financial economics, Financial market, International finance, International economics

## Abstract

Empirical and theoretical research concurs to show that style investing increases return correlations within assets that are classified into the same style. The theoretical model presented in this study addresses the question of how the correlation increases due to style investing depend on market size, and how they respond to economic downturns and to the incidence and awareness of style investing. The results show that correlation distortions caused by style investing are more robust for smaller markets. Further, the effect of style investing on correlations strengthens risk aversion, and hence during downturns. Market awareness, and incidence, of style investing also increase correlation distortions. The model yields closed-form analytical expressions for the correlation distortions caused by style investing, as well as for the effects of changes in risk aversion and in the incidence and awareness of style investing. Given the surge ETF-based style investing over the last two decades, the results have implications for portfolio risk diversification. This study predicts that the ability of risk mitigation through portfolio diversification diminishes particularly for small-market domestic investors as a result of the growing relevance of country-based ETF-based.

## 1 Introduction

Cross-country variation in the average correlation across securities returns has been linked to a variety of explanations, including institutional differences (Morck et al. (2000)), degrees of capital market openness (Li et al. (2004)), lack of transparency at the firm level (Jin and Myers (2006)), limits to arbitrage (e.g., Bris et al. (2007)), and correlated beliefs in firm-level information (David and Simonovska (2016)). This study proposes that style investing provides an additional explanation to the variability in return correlations.

The empirical literature has provided evidence consistent with investors' tendency to follow styles in designing their portfolio (e.g., Kumar (2009)). Style investing, especially in the form of index investing, has been identified as one of the causes of the demand shocks causing correlation distortions from the levels implied by securities' fundamental values (e.g., Greenwood and Thesmar (2011), Anton and Polk (2014)).^1^ Asset correlations are important for risk management as they determine the potential for risk diversification in portfolio management.

The theoretical model of Barberis and Shleifer (Barberis and Shleifer (2003), henceforth, BS), predicts that the correlation between securities grouped into the same style should rise above the level implied by fundamentals, due to correlated shifts in demand caused by the portfolio rebalancing of style investors. These demand shocks are mitigated by the activities of non-style investors (i.e., fundamental traders), who lean against the price deviations caused by style investing.

An increase in within-group return correlations adversely affects investors' ability to mitigate risk. Hence, the implication of the BS model is that style investing weakens the potential for risk mitigation. The theoretical model presented in this study addresses the additional question of whether the extent of this detrimental effect depends on group size.

This study extends the BS model in three directions. The first novelty is that the expectations of non-style investors are explicitly modeled. This added level of complexity yields closed-form analytical expressions for the correlation distortions caused by style investing, on which comparative statics can be performed. A second departure from the BS framework is that fundamental traders are assumed to be aware, if imperfectly, of the impact of the activities of style investors. This feature is realistic, as the popularity of style investing, in all its forms, has been increasing over time.^2^ Lastly, the number of securities included in the style portfolios is allowed to be uneven, whereas BS focus on securities groups of the same numerosity. Their approach is appropriate when evaluating the effect of style investing across asset groupings of similar size, like, for example, US equities and US (liquid) corporate bonds. However, assuming that styles include a similar number of assets is limiting, especially when analyzing the implication of style investing for international financial markets.

Over the last two decades, the surge of country-based investing (e.g., Israel and Maloney (2014), Ben-David et al. (2017)) has resulted in the shuffling of large amounts of wealth among country or region-based portfolios tracking popular market indexes (e.g., the S&P 500 index, MSCI indexes). The increased popularity of country investing has been facilitated by the availability of country or region-based exchange-traded funds (ETFs).^3^ Consistently, many country ETFs are on the list of Top 20 funds, by traded volume. In terms of assets under management, as of the end of 2018, the largest ETFs cover US securities, but runner-up ETFs focus on geographic areas covering several non-US markets.^4^ Popular choices among investors have been country-based ETF for Brazil, Japan, China, Taiwan, India, Hong Kong, Mexico, Germany and South Korea. The number of securities grouped by these ETFs shows significant variation.^5^

This study predicts that the within-style correlation increases caused by group investing are stronger for securities categories including fewer assets. This result has practical implications for risk management for investors of smaller economies. It has been long recognized that investors overinvest in domestic stocks and other domestic assets (e.g., Chan et al. (2005), Ardalan (2019)). Hence, an increase in the correlations among these securities matters for the perspective of portfolio management, as highly correlated within-country returns decrease the scope for domestic portfolio diversification. The results of this study predict that this detrimental effect is stronger for the investors operating in smaller economies. For the portion of their portfolio that is allocated in the domestic market, which is usually large, investors operating in smaller economies have fewer and more correlated assets with which to diversify their risk exposure than investors that are active in broader markets.

Beyond international investing, this study is also relevant to other forms of securities grouping. For instance, industry-based ETFs are also very popular choices with investors. One of the implications of the model is that increases in the incidence of industry-based investing make within-industry diversification harder, and particularly so for the less populated industry sectors. Consistently, the empirical results presented in Chan et al. (2007) show a negative relationship between the average number of companies in each industry (using several GICS classifications) and the within-industry pair-wise correlation of equity excess returns. This study argues that this negative association is due, at least partially, to industry-based investing.

Campbell and Cochrane (1999) have shown that risk aversion increases during downturns, following losses. Building on this insight, another question addressed in this study is how the correlation increases caused by style investing respond to downturns. The prediction is that correlations increase during downturns. Given the strong evidence of investors' home bias, the prediction is thus that style investing reduces the potential for risk mitigation exactly when risk diversification is needed the most, that is during downturns.

In modeling the expectations of fundamental traders, this study also takes into account that non-style investors gauge the impact of style investing, if imprecisely. This feature allows showing that the effect of style investing on correlations is magnified by fundamental traders' awareness of style investing. The intuition is that risk-averse fundamental traders recognize that the activities of style investors cause a higher level of risk, in the form of correlated demand shocks. Their response is thus similar to that triggered by an increase in risk aversion, and it can be understood as a lower willingness to mitigate the distorting effect on prices of the activities of style investors.

As in BS, we show that style investing decreases the correlation across asset groups. Additionally, this study finds that this effect is generally increasing in risk aversion, as well as in the awareness and incidence of style investing. Empirically, the correlation between equities and sovereign bonds have switched sign (from positive, to negative) over the recent decades, as well as the increased severity of the correlation drops during periods of heightened risk aversion (i.e., flight-to-safety episodes, see Baele et al. (2019)) Both these empirical facts are consistent with the extraordinary growth in mutual funds, and more recently ETFs, observed over the same period, as these investment vehicles typically classify equities and sovereign bonds as separate asset classes.

The next section outlines the model. Proofs are in the appendix.

## 2 A model

There are two markets with a different number of securities.^6^ The securities of each market are grouped together by style investors. To fix ideas, one can identify the market with more securities with the US, while the market with fewer assets can be an emerging market covered by a popular country ETF. Assets can be thought of as stocks listed in the respective domestic exchanges, grouped by country ETFs covering the main domestic equity index.^7^

The model is dynamic, and agents decide their allocations simultaneously at the beginning of each period. There are two types of agents: style investors, also called switchers, and fundamental investors. In each period, style investors' demand for either asset group is determined by its past performance relative to the other. In contrast, fundamental traders do not separate securities into styles and invest according to return expectations, which are implied by a factor model. As assumed in BS, fundamental traders are not sufficiently sophisticated to infer how style investors vary their allocations.^8^ Equilibrium prices are yielded by market clearing.

The effect of style investing is measured by gaps between the return correlation implied by equilibrium prices and by securities' fundamentals (i.e., cash-flows). The research questions are whether these correlation gaps are different for groups containing a different number of securities, and how return correlations respond to changes in fundamental traders' risk aversion as well as to the awareness and incidence of style investing.

### 2.1 Assets

There are two asset classes or groups which are indexed by *X* and *Y*, respectively.^9^ Class *X* and *Y* contain $n_{1}$ and $n_{2}$ securities, respectively, in fixed supply. The payoff of the generic risky assets *i* in class *X* and *Y* is a claim to a single principal $D_{i,T}$ payable at the end of the economy *T*. The time-*t* payoff of asset *i* is described by the following sum:

$$D_{i,t}=D_{i,0}+\varepsilon_{i,1}+\ldots +\varepsilon_{i,t}$$

where $D_{i,0}$ and the shock $\varepsilon_{i,t}$ are announced at time 0 and time *t* respectively. The shocks $\varepsilon_{it}$ are zero-mean identically and independently distributed (iid) random variables. The cash-flow shocks follow a linear factor model, and are determined by the realizations of an overarching market factor $f_{Mt}$ summarizing macroeconomic conditions affecting both groups, by class-specific factors, denoted by $f_{Xt}$, and $f_{Yt}$, respectively, and by a security-specific idiosyncratic shocks $f_{it}$. The effects of the factors are combined by time-invariant weights. These are denoted by *M* for global macroeconomic conditions, *S* for the country-specific shock, and *I* for idiosyncratic risk.^10^

For each *t* the cash-flow shocks for securities in *X* and *Y* take the following form:

(1) $$\varepsilon_{it}=\sqrt{M}f_{Mt}+\sqrt{S}f_{Xt}+\sqrt{I}f_{it}\text{ for }i\text{ in }X$$

(2) $$\varepsilon_{jt}=\sqrt{M}f_{Mt}+\sqrt{S}f_{Yt}+\sqrt{I}f_{jt}\text{ for }j\text{ in }Y$$

where, without loss of generality, it is imposed that:

M+S+I=1

All factors have zero-mean and unit variance, and they are iid. The covariance Σ of the cash-flow shocks is:

(3) $$cov\left(\varepsilon_{it},\varepsilon_{jt}\right)=\left\{ \begin{array}{cc} 1\; & \text{for }i=j\\ M+S\; & \text{for }i\neq j\text{ if }i,j\text{ in the same asset class}\\ M\; & \text{for }i\neq j\text{ if }i,j\text{ in different asset class} \end{array}\right.$$

The price of a security *i* at time *t* is denoted by $P_{i,t}$. Price changes between t−1 and *t* are denoted by:

$$\Delta P_{i,t}=P_{i,t}-P_{i,t-1}$$

For simplicity, price changes are refer to as returns. The time-*t* returns of the equally weighted index of the classes are:

(4) $$\Delta P_{X,t}=\frac{\sum_{i\in X}\Delta P_{i,t}}{n_{1}}$$

(5) $$\Delta P_{Y,t}=\frac{\sum_{j\in Y}\Delta P_{j,t}}{n_{2}}$$

### 2.2 Switchers

Style investors, or switchers, invest uniformly in all the securities of each asset class.^11^ They are also subject to extrapolation bias, and modify their holdings in each group on the basis of its relative past performance with respect to the other. For instance, if the average return is higher in *X* than *Y* over the previous period, switchers will sell holdings in *Y* and use the proceeds to fund the acquisitions of long positions in class *X*. Switchers' aggregate demand for assets *i* and *j* are:

(6) $$N_{it}^{S\alpha }=\alpha N_{it}^{S}=\frac{\alpha }{n_{1}}\left[A_{X}+\sum_{k=1}^{t-1}\theta^{t-k}\left(\frac{\Delta P_{Xt-k}-\Delta P_{Yt-k}}{2}\right)\right]\text{ for }i\text{ in }X$$

(7) $$N_{jt}^{S\alpha }=\alpha N_{jt}^{S}=\frac{\alpha }{n_{2}}\left[A_{Y}+\sum_{k=1}^{t-1}\theta^{t-k}\left(\frac{\Delta P_{Yt-k}-\Delta P_{Xt-k}}{2}\right)\right]\text{ for }j\text{ in }Y$$

with

$$N_{it}^{S\alpha }\equiv \frac{\alpha N_{Xt}}{n_{1}}\text{ and }N_{jt}^{S\alpha }\equiv \frac{\alpha N_{Yt}}{n_{2}}$$

where $\alpha N_{Xt}$ and $\alpha N_{Yt}$ are the aggregate demands from switchers for each class, the parameter θ∈(0,1) gives the weights on past realizations, and α>0 is a scaling constant summarizing the incidence of style investing in the economy. The parameter *α* allows investigating how the incidence of style investing influences return correlations. The constant $A_{X}$ and $A_{Y}$ can be interpreted as the long-run average of the holdings in each asset class. As in BS, it is assumed that switchers have sufficient funds to support their asset allocations.

### 2.3 Fundamental traders

Fundamental traders have an exponential utility (CARA), and choose the portfolio $N_{t}^{F}$ in the $n_{1}+n_{2}$ securities:

$$\max_{N_{t}^{F}}E_{t}^{F}\left[-\exp\left[-\gamma \left(W_{t}+N_{t}^{F}\left(P_{t+1}-P_{t}\right)\right)\right]\right]$$

where γ>0 is the risk aversion, $W_{t}$ is wealth at time *t*, and $P_{t}$ is the vector of prices for the $n_{1}+n_{2}$ securities.^12^ The superscript *F* refers to the information set of fundamental traders. Fundamental traders assume normally distributed conditional returns, with return variance matrix $V_{t}$ defined by:

$$V_{t}=var^{F}\left(P_{t+1}-P_{t}\right)$$

so that their holding for each asset *i* satisfies:

(8) $$N_{t}^{F}=\frac{V_{t}^{-1}}{\gamma }\left(E_{t}^{F}\left(P_{t+1}\right)-P_{t}\right)$$

Note that fundamental investors trade more aggressively when current prices are further away from the level implied by their expectations. In this sense, fundamental traders lean against the price deviations caused by switchers.

Securities are in fixed supply, so that:

$$P_{i,t}=E^{F}\left(P_{it+1}\right)-\gamma V_{t}\left(N_{t}^{F}\right)$$

where:

$$N_{t}^{F}=Q-N_{t}^{S\alpha }$$

with *Q* being the vector of supplies $Q_{i}$, where $N_{t}^{S\alpha }$ is the vector of $N_{it}^{S\alpha }$ and $N_{jt}^{S\alpha }$ for i∈X and j∈Y. Fundamental traders base their expectations on the final dividends $D_{T}$ for the $n_{1}+n_{2}$ assets. In vector notation:

$$E_{T-1}^{F}\left(P_{T}\right)=E_{T-1}^{F}\left(D_{T}\right)=D_{T-1}$$

and prices may be obtained by backward substitution:

$$P_{t}=D_{t}-\gamma V_{t}\left(Q-N_{t}^{S\alpha }\right)-E_{t}^{F}\left(\sum_{k=1}^{T-t-1}\gamma V_{t+k}\left(Q-N_{t+k}^{S\alpha }\right)\right)$$

Fundamental traders not only do recognize the factor structure of the shocks affecting cash-flows, but they also recognize the existence of switchers, if indirectly. As in BS, I assume that fundamental traders are not sufficiently sophisticated to figure out how switchers determine their allocation. However, differently from BS, I assume that fundamental traders model style-based demand shocks by two additional group-specific risk factors, $f_{Ht}$ and $f_{Kt}$, which have unit variance and are serially uncorrelated. The institutional factors are negatively correlated, so that $corr\left(f_{Ht},f_{Kt}\right)=-1$, which models fundamental traders' intuition of the opposite effects of switchers' demand shocks on each asset class. The lack of correlation of $f_{Ht}$ and $f_{Kt}$ with the factors determining the cash-flows is consistent with switchers' activities being based on past prices, given that these factors determining the shocks are serially uncorrelated. To interpret, $f_{Ht}$ and $f_{Kt}$ are institutional factors, in the sense that they capture fundamental traders' awareness of style investing. Fundamental traders assume the return generating process:

(9) $$\Delta P_{it}^{F}=\sqrt{M}f_{Mt}+\sqrt{S}f_{Xt}+\sqrt{H\alpha }f_{Ht}+\sqrt{I}f_{it}\text{ for }i\text{ in }X$$

(10) $$\Delta P_{jt}^{F}=\sqrt{M}f_{Mt}+\sqrt{S}f_{Yt}+\sqrt{H\alpha }f_{Kt}+\sqrt{I}f_{jt}\text{ for }j\text{ in }Y$$

The parameter *H* modules the intensity of fundamental traders' perception of style investing in the economy. The realizations of the institutional factors are further scaled by the parameter *α*, which also governs the presence of switchers in the economy.^13^ Note that the higher is the incidence of style investors, the stronger is the effect of the institutional factors. Under these assumptions, the fundamental traders' variance matrix is time-invariant and is:

(11) $$V=cov^{F}\left(\Delta P_{it+1}^{F},\Delta P_{jt+1}^{F}\right)=\left\{ \begin{array}{cc} 1+\alpha H\; & \text{for }i=j\\ M+S+\alpha H\; & \text{for }i\neq j\text{ if }i,j\text{ same asset class}\\ M-\alpha H\; & \text{for }i\neq j\text{ if }i,j\text{ different asset class} \end{array}\right.$$

### 2.4 Prices and returns

Beyond proxying the activities of switchers with the inclusion of the institutional factors, fundamental traders consider switchers zero-mean supply shocks, so their expectation for switchers' demand is time-invariant, and it is denoted by ${\bar{N}}^{S\alpha }$. Formally, we have:

$$E_{t}^{F}\left(N_{t+k}^{S\alpha }\right)={\bar{N}}^{S\alpha }$$

Given these expectations, prices simplify to:

$$P_{t}=D_{t}-\gamma V\left(Q-N_{t}^{S\alpha }\right)-\left(T-1-t\right)\gamma V\left(Q-{\bar{N}}^{S\alpha }\right)$$

Dropping non stochastic terms yields:

(12) $$P_{t}=D_{t}+\gamma VN_{t}^{S\alpha }$$

Proposition 1 *The equilibrium prices are*

(13) $$\Delta P_{it+1}=\varepsilon_{it+1}+\gamma \Delta N_{Xt+1}^{S\alpha }A_{1}\;\text{for}\;i\;\text{in}\;X$$

(14) $$\Delta P_{jt+1}=\varepsilon_{jt+1}+\gamma \Delta N_{Yt+1}^{S\alpha }A_{2}\;\text{for}\;j\;\text{in}\;Y$$

*where*

(15) $$A_{1}=S+2H\alpha +\frac{I}{n_{1}}>0$$

(16) $$A_{2}=S+2H\alpha +\frac{I}{n_{2}}>0$$

Note that when there are no switchers in the economy (i.e., when α=0), then returns are determined solely by fundamentals. The next proposition spells out the sign of the return correlations.

Proposition 2 *Let* i,j∈X *and* h,k∈Y*. Then*

$$cov\left(\Delta P_{it+1},\Delta P_{jt+1}\right)>0$$

$$cov\left(\Delta P_{ht+1},\Delta P_{kt+1}\right)>0$$

*and*

$$cov\left(\Delta P_{it+1},\Delta P_{jt+1}\right)<0\;\text{for}\;v>v_{0}=\frac{M}{\gamma^{2}\alpha^{2}A_{1}A_{2}}$$

*where:*

(17) $$v=var\left(\Delta N_{Xt+1}^{S}\right)$$

The proposition shows that returns are positively correlated within asset groups, which is expected given the cash-flow dynamics and the investment activities of switchers. Further, for returns to be negatively correlated across asset groups, the shocks to switchers' demand must be sufficiently large to outweigh the positive correlation caused by the exposure to the common factor $f_{Mt}$.

## 3 Correlation gaps

Due to the activities of switchers, within-group return correlations are different from the correlation levels of the underlying cash-flows. The spread between the return correlation and the correlation implied by cash-flows is termed the (intra-class) correlation gap. Formally, for group *X* we have:

(18) $$GAP_{n_{1}}\left(i,j\right)=corr\left(\Delta P_{it+1},\Delta P_{jt+1}\right)-corr\left(\varepsilon_{it+1},\varepsilon_{jt+1}\right)\text{ for any }i,j\in X$$

The analogous expression defines the correlation gap $GAP_{n_{2}}$ for group *Y*.

In the framework proposed by BS, style investing entails higher levels of correlations than those implied by fundamentals. The result is confirmed by the next proposition. However, as fundamental traders' expectations are explicitly modeled, this study additionally yields the analytical expressions of the correlation gaps, which will allow us to evaluate the effects of shocks to risk aversion and awareness and incidence of style investing.

Proposition 3 *Within-group returns are more correlated than the underlying cash-flows, and*

(19) $$GAP_{n_{1}}\left(i,j\right)=GAP_{n_{1}}=\frac{I\gamma^{2}v\alpha^{2}A_{1}^{2}}{1+\gamma^{2}v\alpha^{2}A_{1}^{2}}>0$$

(20) $$GAP_{n_{2}}\left(h,k\right)=GAP_{n_{2}}=\frac{I\gamma^{2}v\alpha^{2}A_{2}^{2}}{1+\gamma^{2}v\alpha^{2}A_{2}^{2}}>0$$

*where* i,j∈X *and* h,k∈Y*.*

Note that if there are no switchers in the economy (i.e., if α=0) the correlation gaps are zero.

The correlation gaps are measures of correlation distortions for returns at a given moment in time, say time *t* for correlations in the period between *t* and t+1. Hence, the evaluation of the effect of changes in the exogenous parameters on $GAP_{n_{1}}$ and $GAP_{n_{2}}$ is performed conditionally on the equilibrium levels reached at time *t*, and for small changes. In particular, shocks in exogenous variables occurring after the prices $P_{t}$ have been determined do not affect the demand levels of switchers $N_{Xt+1}^{S}$, and $N_{Yt+1}^{S}$ (and thus *v*), as these depend on prices up to time *t*.^14^ The next proposition identifies the effects of the numerosity of the asset class, risk aversion, awareness and incidence of style investing on the correlation gaps.

Proposition 4 *The intra-class correlation gaps* $GAP_{n_{1}}$ *and* $GAP_{n_{2}}$ *decline as the number of securities increases; increase in the risk aversion of fundamental traders; increase the more switchers are in the economy; increase in fundamental traders' awareness of style investing.*

The intuition for the effect of the number of assets in the market is that a larger number of securities dilutes the effect of the trades of switchers. Hence, when the number of securities becomes larger, the deviation from the fundamental level of correlation diminishes. The effect of an increase in the risk aversion of fundamental traders is instead to increase correlation distortions. The reason is that fundamental traders lean against the activities of switchers, which they regard as supply shocks. More risk-averse fundamental traders invest less in both classes of risky assets and thus they mitigate less the effect of the activities of switchers on return correlations. Naturally, the more prevalent is style investing, the stronger are correlation distortions.^15^ The results also show that the more aware fundamental traders become of style investing (parameter *H*), the stronger the correlation distortions. From fundamental traders' perspective, higher levels of *H* increase the risk level of returns, and thus deter their participation.

Awareness of style investing may be a function of its incidence, so that the parameter *H* is an increasing function of *α*. Adding this assumption does not alter the conclusion that more incidence (and thus awareness) of style investing increases the correlation gaps, as shown in the proof of Proposition 4.

### 3.1 Correlation gaps spread and relative market size

As shown in Proposition 3, the correlation distortions are not equal across the two asset groups. We can thus define the spread between the correlation gaps for *X* and *Y* to assess whether style investing has a different effect on return correlations in asset groups that include a different number of securities. The spread between the correlation gaps of *X* and *Y* is denoted by ΔGAP, and is defined as follows:

(21) $$\Delta GAP=GAP_{n_{2}}-GAP_{n_{1}}$$

The following proposition evaluates the sign of the correlation distortion spread ΔGAP defined in (21). To simplify the exposition, we can identify *X* as the group with the highest number of securities, so that $n_{1}>n_{2}$. Again, one can think of group *X* as the US equity market and group *Y* as an emerging country equity market, where country equity ETFs allow country based style investing.

Proposition 5 *Let* $n_{1}=sn_{2}$*, with* s>1*. Then*

ΔGAP>0

*Moreover,*

$$\frac{\partial \Delta GAP}{\partial s}>0$$

*Further, if* $n_{1}=n_{2}$ *then* ΔGAP=0*.*

These results indicate that when style investing involves two groups of assets with different numerosity, style investing does not affect correlations uniformly. Rather, correlation increases are stronger in the asset group including the lowest number of securities. The intuition behind this result is that style investing affects correlation less when switchers demand shocks spread over a large number of securities, as shown in Proposition 4.^16^

The following proposition presents some comparative statics results for ΔGAP. The results are presented in terms of the variance of switchers' demand shocks (i.e., the variable *v*, defined in equation (17)).^17^

Proposition 6 *Let* $n_{1}=sn_{2}$*, with* s>1*, then*

$$sgn\left(\frac{\partial \Delta GAP}{\partial v}\right)=sgn\left(v_{1}-v\right)$$

$$sgn\left(\frac{\partial \Delta GAP}{\partial \gamma }\right)=sgn\left(v_{1}-v\right)sgn\left(\frac{\partial \Delta GAP}{\partial \alpha }\right)=sgn\left(v_{1}-v\right)sgn\left(\frac{\partial \Delta GAP}{\partial H}\right)=sgn\left(v_{1}-v\right)$$

*where*

(22) $$v_{1}=\frac{1}{\alpha^{2}\gamma^{2}A_{1}A_{2}}$$

*Further, for sufficiently large values of s, we have* $v_{1}>v$*.*

Comparative statics on ΔGAP yields results that depend on the level of the volatility of the shocks of switchers' demand (i.e., the variable *v*), which, in turn, depends on the past realizations of the cash-flow shocks. The findings are nuanced. For low levels of *v*, increases in the volatility of switchers' demand shocks, higher risk aversion (which decreases the participation of fundamental traders), more switchers, and more awareness of style investing (which adds to the risk level of the cash-flows and thus deters risk averse fundamental traders' participation) all tend to exacerbate the effects of style investing. These effects make the correlations distortions $GAP_{n_{2}}$ in class *Y* increasingly more steeply than the correlation distortion $GAP_{n_{1}}$ in class *X*.

When the fluctuations of switchers' demand are large, the spreads between the correlation gaps decreases, which is consistent with fundamental investor leaning in more aggressively to counter the effects of style investing. This response dampens the effects of increases in *γ*, *H* and *α* (which tend to reinforce the role of style investing in determining prices) and causes a trend toward convergence of the correlation gaps $GAP_{n_{1}}$ and $GAP_{n_{2}}$. Both gaps continue to increase in risk aversion, and in the awareness and incidence of style investing, as noted in Proposition 4. However, large fluctuations in switchers' demand cause these effects to converge in the two classes.^18^

The threshold $v_{1}$ displayed in equation (22) is an increasing and unbounded function of *s*. In contrast, the fluctuations of the switchers' positions are bounded by the fixed supply of securities. Hence for sufficiently large values of *s* the variance *v* will always fall below the threshold $v_{1}$. Hence, when the disparity between the numerosity of the asset groups is sufficiently large, the effect of increases in risk aversion, and in the incidence and awareness of style investing, is to make correlation distortions larger in the groups with fewer securities.

### 3.2 Inter-class correlation gaps

ETF providers refer to international diversification as one of the key advantages of investing in this type of products, a claim that is at least partially supported by empirical evidence (Cao et al. (2017)). The predictions presented hereafter appear to lend validity to their assessment. Define the inter-class correlation gap as the difference between the correlations of asset returns and of cash-flows, for assets in different groups. Formally,

(23) $$interGAP\left(i,j\right)=corr\left(\Delta P_{it+1},\Delta P_{jt+1}\right)-corr\left(\varepsilon_{it+1},\varepsilon_{jt+1}\right)\text{for any }i,j\text{ in different asset classes}$$

The following proposition shows that style investing results in a decline of the correlation across asset classes, regardless of the disparity in the numerosity of each asset class.

Proposition 7 *Let* $n_{1}=sn_{2}$ *with* s>0*. The correlation between assets in X and assets in Y is lower than the level implied by the underlying cash-flows, so that:*

interGAP<0

Style-based financial products (e.g., country-ETF) are typically marketed to investors as a tool for risk diversification. Hence, it is interesting to explore how risk aversion affects the correlation across asset classes. In the simplified case in which the classes *X* and *Y* include an equal number of securities, we can unambiguously identify the direction of the effects of exogenous parameters on the inter-class correlation distortion.^19^

Proposition 8 *If* $n_{1}=n_{2}$*, the correlation distortion across asset classes* interGAP *is negative and its absolute value increases in the risk aversion of fundamental traders; increases the more switchers are in the economy; increases in fundamental traders' awareness of style investing.*

The implication is that returns are less correlated than warranted by fundamentals across style groupings when style investing is more prevalent and acknowledged, and when risk aversion is large. Proposition 8 offers a counterfactual scenario to evaluate whether asset groupings including about the same number of securities (e.g., US and pan-European ETFs) result in a reduction of risk for international investors. If two asset groups of about equal numerosity are really separate investment styles, then in periods of heightened risk aversion, style investing should cause a decrease in their correlation that goes beyond the level implied by fundamental valuations.

When the numerosity of the classes is different, the effects of the exogenous parameters depend on the volatility of switchers' demand shocks (i.e., variable *v*) in a non-trivial way. Once more, the effects of risk aversion, and of the incidence and relevance of style investing are as those documented for the case of equal numerosity (e.g., Proposition 8) for low levels of *v* or when the disparity in numerosity (i.e., parameter *s*) is very large. However, high levels of prices fluctuations might end up reversing the sign of these marginal effects (but not of the interGAP), as fundamental investors trade more aggressively when past prices strongly deviate from their expectations. The associated analytical results are similar to the ones explored for within-class correlation ΔGAP and are left unreported.

As already cautioned by BS, one should be careful in concluding that style investing has univocal predictions for the correlation between two asset groups. The caveat is that styles might overlap. Hence, if for one style two securities are grouped together, for another they might be included in different style portfolios. For example, the equities of France and Germany might be grouped together in an European investment fund, but they might be included in two separate country-based ETFs. Thus the effect of style investing on across-groups correlations should be qualified in empirical investigations to ascertain to which style a security mostly belongs.

## 4 Conclusions

The traditional finance view suggests that securities returns are mainly driven by underlying fundamental values. However, a large body of evidence shows that prices may move together for reasons that appear to be unrelated to fundamentals, with demand shocks explaining a substantial part of price comovements across securities. Style investing, especially in the form of index investing, is one of the causes of these demand shocks (e.g., Greenwood and Thesmar (2011), Anton and Polk (2014)).

This study shows that the within-group correlation distortions due to style investing are stronger for less populated asset groups. Given the strong evidence of domestic bias in investors' allocations, country-based style investing is thus particularly detrimental for the risk diversification potential of small markets' domestic investors. With some caveats, this effect is, unfortunately, more marked when diversification is needed the most, that is during downturns, when risk aversion rises. The reduction in the potential of risk diversification has, of course, to be balanced with a beneficial increase in market liquidity and with the higher informational efficiency brought upon by country ETFs.

This study also finds that the awareness and incidence of style investing raise within-class correlations. Hence, the results support the argument that the surge in country ETFs and their increased popularity are channels through which style investing affects within-country return correlations, and thus investment outcomes of domestic investors.

## 5 Appendix

Proof of Proposition 1 The variance *V* defined by expression (11) is a block matrix with:

$$V=\left(\begin{array}{cc} A\; & B\\ B^{\prime }\; & C \end{array}\right)$$

where *A* and *C* are $n_{1}\times n_{1}$ and $n_{2}\times n_{2}$ matrices, respectively, with diagonal values equal to 1+αH and off-diagonal entries equal to M+S+αH. The matrix *B* is a $n_{1}\times n_{2}$ matrix with all the elements equal to M−αH. Recalling equation (12), for the return $\Delta P_{it+1}$ with i∈X we have:

$$\Delta P_{it+1}=\varepsilon_{it+1}+\gamma R_{i}\Delta N_{t+1}^{S\alpha }$$

where $R_{i}$ is raw *i* of *V*, with $i\leq n_{1}$. As $\Delta N_{Xt+1}^{S\alpha }=-\Delta N_{Yt+1}^{S\alpha }$, we have:

$$R_{i}\Delta N_{t+1}^{S\alpha }=\frac{\Delta N_{Xt+1}^{S\alpha }}{n_{1}}\left(1+\alpha H+\left(n_{1}-1\right)\left(M+S+\alpha H\right)\right)\;+\frac{\Delta N_{Yt+1}^{S\alpha }}{n_{2}}n_{2}\left(M-\alpha H\right)=\frac{\Delta N_{Xt+1}^{S\alpha }}{n_{1}}\left(\left(S+2H\alpha \right)n_{1}+I\right)=\frac{\Delta N_{Xt+1}^{S\alpha }}{n_{1}}A_{1}$$

which yields the expression (13). Analogously, for row *j* with $j>n_{1}$ we have

$$R_{j}\Delta N_{t+1}^{S\alpha }=\frac{\Delta N_{Xt+1}^{S\alpha }}{n_{1}}\left(n_{1}\left(M-\alpha H\right)\right)\;+\frac{\Delta N_{Yt+1}^{S\alpha }}{n_{2}}\left(\left(n_{2}-1\right)\left(M+S+\alpha H\right)+1+\alpha H\right)=\frac{\Delta N_{Yt+1}^{S\alpha }}{n_{2}}\left(\left(S+2H\alpha \right)n_{2}+I\right)$$

which obtains expression (14). □

Proof of Proposition 2 Using the expression of the returns in (13), we calculate the return correlation for assets *i* and *j* in *X*. Note that the change in the position of switchers depends on past prices, and it is, therefore, uncorrelated with current cash-flow shocks. As $\Delta N_{Xt+1}^{S\alpha }=-\Delta N_{Yt+1}^{S\alpha }$, note also that:

$$var(\Delta N_{Xt+1}^{S\alpha })=var(\Delta N_{Yt+1}^{S\alpha })=\alpha^{2}v$$

Recalling equation (3), we find:

(24) $$var\left(\Delta P_{i,t+1}\right)=1+\gamma^{2}A_{1}^{2}\alpha^{2}v\text{ with }i\in X$$

(25) $$var\left(\Delta P_{j,t+1}\right)=1+\gamma^{2}A_{2}^{2}\alpha^{2}v\text{ with }j\in X$$

and thus:

(26) $$corr\left(\Delta P_{it+1},\Delta P_{jt+1}\right)=\frac{\left(M+S\right)+\gamma^{2}v\alpha^{2}A_{1}^{2}}{1+\gamma^{2}\alpha^{2}A_{1}^{2}v}>0$$

Now let i∈X and j∈Y. Note that the changes in switchers' demands, namely, $\Delta N_{Xt+1}^{S\alpha }$ and $\Delta N_{Yt+1}^{S\alpha }$ are symmetric, so that $\Delta N_{Yt+1}^{S\alpha }$ equals $-\Delta N_{Xt+1}^{S\alpha }$. Hence, we have:

(27) $$cov\left(\Delta N_{Xt+1}^{S\alpha },\Delta N_{Yt+1}^{S\alpha }\right)=-\alpha^{2}v.$$

Thus,

(28) $$corr\left(\Delta P_{it+1},\Delta P_{jt+1}\right)=\frac{M-\gamma^{2}\alpha^{2}vA_{1}A_{2}}{\left(1+\gamma^{2}\alpha^{2}A_{1}^{2}v\right)\left(1+\gamma^{2}\alpha^{2}A_{2}^{2}v\right)}.$$

Note that *M* is the cash-flow shocks correlation across asset groups, so that the return correlation across *X* and *Y* is negative only when

$$M<\gamma^{2}\alpha^{2}vA_{1}A_{2}$$

which is zero if α=0. □

Proof of Proposition 3 From Proposition 2 and equation (26) the intra-class correlation gap for class *X* is:

$$GAP_{n_{1}}=\frac{\left(M+S\right)+\gamma^{2}\alpha^{2}vA_{1}^{2}}{1+\gamma^{2}\alpha^{2}A_{1}^{2}v}-\left(M+S\right).$$

A bit of algebra yields equation (19). An analogous result holds for class *Y*. □

Proof of Proposition 4 Taking the derivative with respect to $n_{1}$, *γ*, *α* and *H* of equations (19) and (20) shows the monotonic trends. Note that

$$\frac{\partial GAP_{n_{1}}}{\partial A_{1}}=\frac{2v\alpha^{2}\gamma^{2}IA_{1}}{{\left(v\alpha^{2}\gamma^{2}A_{1}^{2}+1\right)}^{2}}>0$$

and, recalling the expression of $A_{1}$ we have (15). Hence

$$\frac{\partial GAP_{n_{1}}}{\partial n_{1}}=\frac{\partial GAP_{n_{1}}}{\partial A_{1}}\frac{\partial A_{1}}{\partial n_{1}}=\frac{\partial GAP_{n_{1}}}{\partial A_{1}}\left(-\frac{I}{n_{1}^{2}}\right)<0\;\frac{\partial GAP_{n_{1}}}{\partial \gamma }=\frac{2v\alpha^{2}\gamma IA_{1}^{2}}{{\left(v\alpha^{2}\gamma^{2}A_{1}^{2}+1\right)}^{2}}>0\;\frac{\partial GAP_{n_{1}}}{\partial H}=\frac{\partial GAP_{n_{1}}}{\partial A_{1}}\frac{\partial A_{1}}{\partial H}=\frac{\partial GAP_{n_{1}}}{\partial A_{1}}2\alpha >0\;\frac{\partial GAP_{n_{1}}}{\partial \alpha }=\frac{\partial GAP_{n_{1}}}{\partial A_{1}}\frac{\partial A_{1}}{\partial \alpha }+{\frac{\partial GAP_{n_{1}}}{\partial \alpha }|}_{A_{1}}=\frac{\partial GAP_{n_{1}}}{\partial A_{1}}2H+\frac{2v\alpha \gamma^{2}IA_{1}^{2}}{{\left(v\alpha^{2}\gamma^{2}A_{1}^{2}+1\right)}^{2}}>0$$

If *H* is a differentiable increasing function of *α*, then

$$\frac{\partial GAP_{n_{1}}}{\partial \alpha }=\frac{\partial GAP_{n_{1}}}{\partial A_{1}}\frac{\partial A_{1}}{\partial \alpha }+\frac{\partial GAP_{n_{1}}}{\partial A_{1}}\frac{\partial A_{1}}{\partial H}\frac{\partial H}{\partial \alpha }+{\frac{\partial GAP_{n_{1}}}{\partial \alpha }|}_{A_{1}}>0$$

Analogous results hold for class *Y*. □

Proof of Proposition 5 With $n_{1}=sn_{2}$ the expression of $A_{1}$ in (15) modifies to:

(29) $$A_{1}=S+2H\alpha +\frac{I}{sn_{2}}$$

Recalling the expression of the correlation gaps in (19) and (20), after a few calculations we have:

$$\Delta GAP=-\frac{v\alpha^{2}\gamma^{2}I\left(A_{1}^{2}-A_{2}^{2}\right)}{\left(v\alpha^{2}\gamma^{2}A_{1}^{2}+1\right)\left(v\alpha^{2}\gamma^{2}A_{2}^{2}+1\right)}$$

Substituting the expression in (29) for $A_{1}$, we obtain that

(30) $$sgn(A_{1}^{2}-A_{2}^{2})=sgn(1-s)$$

which yields the conclusion on the sign of ΔGAP. As the constant *s* only enters the expression of the spread in correlation distortions through $A_{1}$, we get:

$$\frac{\partial \Delta GAP}{\partial s}=\frac{\partial \Delta GAP}{\partial A_{1}}\frac{\partial A_{1}}{\partial s}=\left(-\frac{2v\alpha^{2}\gamma^{2}IA_{1}}{{\left(v\alpha^{2}\gamma^{2}A_{1}^{2}+1\right)}^{2}}\right)\left(-\frac{I}{s^{2}n_{2}}\right)>0$$

 □

Proof of Proposition 6 Note that

$$\frac{\partial \Delta GAP}{\partial v}=\alpha^{2}\gamma^{2}\frac{I\left(v^{2}\alpha^{4}\gamma^{4}A_{1}^{2}A_{2}^{2}-1\right)\left(A_{1}^{2}-A_{2}^{2}\right)}{{\left(v\alpha^{2}\gamma^{2}A_{1}^{2}+1\right)}^{2}{\left(v\alpha^{2}\gamma^{2}A_{2}^{2}+1\right)}^{2}}$$

so that

$$sgn\left(\frac{\partial \Delta GAP}{\partial v}\right)=sgn\left(1-v^{2}\alpha^{4}\gamma^{4}A_{1}^{2}A_{2}^{2}\right)$$

Consistently, we find:

$$\frac{\partial \Delta GAP}{\partial \alpha }=\frac{4Hv\alpha^{2}\gamma I\left(A_{1}^{2}-A_{2}^{2}\right)}{{\left(v\alpha^{2}\gamma^{2}A_{1}^{2}+1\right)}^{2}{\left(v\alpha^{2}\gamma^{2}A_{2}^{2}+1\right)}^{2}}\left(v^{2}\alpha^{4}\gamma^{4}A_{1}^{2}A_{2}^{2}-1\right)\frac{\partial \Delta GAP}{\partial H}=\frac{4v\alpha^{3}\gamma I\left(A_{1}^{2}-A_{2}^{2}\right)}{{\left(v\alpha^{2}\gamma^{2}A_{1}^{2}+1\right)}^{2}{\left(v\alpha^{2}\gamma^{2}A_{2}^{2}+1\right)}^{2}}\left(v^{2}\alpha^{4}\gamma^{4}A_{1}^{2}A_{2}^{2}-1\right)\frac{\partial \Delta GAP}{\partial \gamma }=\frac{2v\alpha^{2}\gamma I\left(A_{1}^{2}-A_{2}^{2}\right)}{{\left(v\alpha^{2}\gamma^{2}A_{1}^{2}+1\right)}^{2}{\left(v\alpha^{2}\gamma^{2}A_{2}^{2}+1\right)}^{2}}\left(v^{2}\alpha^{4}\gamma^{4}A_{1}^{2}A_{2}^{2}-1\right)$$

From equation (30), when s>1 then $A_{1}^{2}-A_{2}^{2}<0$. Hence, for s>1, we have:

$$sgn\left(\frac{\partial \Delta GAP}{\partial H}\right)=sgn\left(\frac{\partial \Delta GAP}{\partial \alpha }\right)=sgn\left(\frac{\partial \Delta GAP}{\partial \gamma }\right)=sgn\left(1-v\alpha^{2}\gamma^{2}A_{1}A_{2}\right)$$

Define the function ϕ(s) as

$$\phi \left(s\right)=\frac{1}{\alpha^{2}\gamma^{2}A_{1}A_{2}}>0$$

where only $A_{1}$ is a function of *s* as in (29). Note that *ϕ* is monotonically increasing and unbounded as a function of *s*. Given the model parameters (s,α,γ,S,H), denote by $v_{1}$ the (unique) level of *v* satisfying

$$v_{1}=\phi \left(s\right)$$

with $v_{1}\left(s,\alpha ,\gamma ,S,H\right)$. Then the partial derivatives of ΔGAP are positive for $v<v_{1}$. Note that $v_{1}$ is increasing in *s*. To see this, note that the scaling factor *s* enters $v_{1}$ only through $A_{1}$, and $A_{1}$ is decreasing in *s*. As its maximum, the variation in the position of switchers is $2Q_{i}$ for security *i*, where $Q_{i}$ is the total supply of security *i*. Hence, the variable *v* is necessarily bounded. Thus, for *s* sufficiently large we have $v<v_{1}$ and thus the partial derivatives of ΔGAP are positive. □

Proof of Proposition 7 From Proposition 2 and equation (28), the inter-correlation gap is:

$$interGAP=\frac{M-\gamma^{2}\alpha^{2}vA_{1}A_{2}}{\left(1+\gamma^{2}\alpha^{2}A_{1}^{2}v\right)\left(1+\gamma^{2}\alpha^{2}A_{2}^{2}v\right)}-M$$

and, after some calculations, we have:

(31) $$interGAP=\frac{-v\alpha^{2}\gamma^{2}\left(Mv\alpha^{2}\gamma^{2}A_{1}^{2}A_{2}^{2}+MA_{1}^{2}+A_{1}A_{2}+MA_{2}^{2}\right)}{\left(v\alpha^{2}\gamma^{2}A_{1}^{2}+1\right)\left(v\alpha^{2}\gamma^{2}A_{2}^{2}+1\right)}<0$$

 □

Proof of Proposition 8 Assume $n_{1}=n_{2}$, then $A_{1}=A_{2}$, then taking the derivative with respect to *γ*, *α* and *H* of equation (31), we have:

$$interGAP=-\frac{\gamma^{2}v\alpha^{2}A_{2}^{2}\left(1+M\right)}{1+\gamma^{2}\alpha^{2}vA_{2}^{2}}<0$$

and

$$\frac{\partial interGAP}{\partial \gamma }=-\frac{2v\alpha^{2}\gamma A_{2}^{2}\left(M+1\right)}{{\left(v\alpha^{2}\gamma^{2}A_{2}^{2}+1\right)}^{2}}<0$$

and using

$$\frac{\partial interGAP}{\partial A_{2}}=-\frac{2v\alpha^{2}\gamma^{2}A_{2}\left(M+1\right)}{{\left(v\alpha^{2}\gamma^{2}A_{2}^{2}+1\right)}^{2}}$$

we get

$$\frac{\partial interGAP}{\partial H}=-\frac{\partial interGAP}{\partial A_{2}}2\alpha <0\;\frac{\partial interGAP}{\partial \alpha }=-\frac{\partial interGAP}{\partial A_{2}}2H<0$$

 □

## Declarations

### Author contribution statement

V. Galvani: All aspects of the contributions are from the author.

### Funding statement

This research did not receive any specific grant from funding agencies in the public, commercial, or not-for-profit sectors.

### Competing interest statement

The authors declare no conflict of interest.

### Additional information

No additional information is available for this paper.

## Footnotes

1 Most of these studies focus on membership changes for country-based equity indexes. An exception is Wahal and Yavuz (2013). Excess comovement has been documented for S&P500 index additions and deletions (Vijh (1994), Barberis et al. (2005)), for changes in S&P500 value and growth indexes (Boyer (2011)), for changes in the Nikkei 225 index (Greenwood and Sosner (2007)), for changes in UK, Japanese, and other national indexes (Mase (2008), Greenwood (2007), Claessens and Yafeh (2013)), among others. These assessment are complicated by the need to control for changes in fundamental return drivers, which might be imprecisely measured (Chen et al. (2016)).

2 Demand for products based on style investing might arise from categorization due to limitations in attention span (e.g., Peng and Xiong (2006)) or in response to both limited cognition and limited data (Al-Najjar and Pai (2014)).

3 For instance, early contributions (e.g., Bekaert and Urias (1999)) already noted that without low-cost investing vehicles able to replicate country indexes, investing in emerging was unlikely to offer significant diversification benefits. According to Miffre (2007), international country ETFs offer such investment opportunity.

4 Source: Morningstar.

5 Smaller economies are often bundled together by international style investors, but the weights on each country are fairly constant over time. Hence, demand shocks for an international fund covering the securities of a set of countries transmit to shocks for the demand of the securities in each country (Jotikasthira et al. (2012), Brooks and Del Negro (2005)).

6 The model setting is that of a dynamic discrete-time model with finite horizon, multiple assets and two agent types.

7 Most country ETFs offer currency hedging. Hence, this study abstracts from the effects of the exchange rate and of reserve currencies. This restriction allows focusing on the effect of differences in market size alone.

8 As noted in BS, assuming that fundamental traders fully understand how style investors' operate opens the door to “price run dynamics”.

9 This study focuses on styles or investment groups for which membership is known at the time securities are issued. Membership is also time-invariant.

10 The notation for the weights follows Barberis and Shleifer (2003), to foster comparability. In their study, *M* stands for market, *S* for style, and *I* for idiosyncratic risk.

11 Allowing for unequal weights would complicate the exposition without yielding additional insights.

12 All vectors and matrices relative to the $n_{1}+n_{2}$ securities are indexed with the $n_{1}$ securities of *X* followed by the $n_{2}$ securities of *Y*, always listed in the same order.

13 Adding some iid measurement error on fundamental traders' evaluation of *α* does not alter this paper's conclusions.

14 For ease of notation, the variance *v* is not indexed on time.

15 The results in Proposition 4 yield empirically testable hypotheses for studies aiming to explain asset correlations in international markets. The volatility and levels of switchers' demand can be measured by the variance and levels of assets under management of country ETFs. Risk aversion can be measured by implied volatility or its decompositions (e.g., Bekaert et al. (2013)). Awareness of style investing is harder to measure, though measures of news coverage of ETFs might provide a suitable proxy.

16 To gain a sense of the range of parameter *s*, we can refer to the empirical literature. Using the number of equities in developed countries reported in Bekaert et al. (2009), and allowing the US equity market to represent class *X*, the parameter *s* ranges from 2.66 (Japan) to 95.54 (Ireland). For developing countries, the scalar *s* tends to be larger.

17 Note that, for a given time *t*, the variance *v* of switchers' demand changes is not stochastic.

18 This convergence occurs for higher levels of *v* when the disparity in numerosity in *X* and *Y* is larger. See proof of Proposition 5.

19 The case $n_{1}=n_{2}$ extends the framework of BS by accounting for the incidence and awareness of style investing. Further BS do not explore the effects of changes in risk aversion *γ*.
